# Supplementary material for: Association between Iron Status and Survival in Patients on Chronic Hemodialysis
Source: Nutrients. 2023 May 31;15(11):2577. doi: 10.3390/nu15112577 (PMC10255805; doi:10.3390/nu15112577)
Supplement: Supplementary file 1 [file nutrients-15-02577-s001.zip › nutrients-2385489-supplementary.pdf]

Supplementary Material

Table S1. Medication types and Health Insurance Review and Assessment Service codes

Table S2. ICD-10 codes of Charlson Comorbidity Index

Table S3. Cox regression analyses using Groups 2 or 3 as reference groups

Table S4. Summary of previous studies evaluating the association between iron status and mortality in patients on HD using large sample sizes ($n \geq 10{,}000$)

Table S1. Medication types and Health Insurance Review and Assessment Service codes

Medications	Codes
**Acepril**	104201ATB, 104202ATB
**Amlodipine**	495901ATB, 459802ACH, 483201ATB, 486501ATB, 107601ATB, 107601ATD, 459801ACH, 459801ATB, 459901ATB, 464601ATB, 470801ATB, 476201ATB, 479701ATB, 483202ATB, 486502ATB, 107602ATB, 107602ATD, 470802ATB
**Amlodipine+Atorvastatin**	614500ATB, 472300ATB472400ATB472500ATB518900ATB
**Amlodipine+Losartan+Chlorthalidone**	662800ATB, 662900ATB, 663000ATB
**Amlodipine+Losartan+Rosuvastatin**	663900ATB, 664000ATB, 664100ATB, 664200ATB, 664300ATB, 664400ATB
**Amlodipine+Olmesartan+Rosuvastatin**	677300ATB, 677400ATB, 677500ATB, 677600ATB
**Amlodipine+Rosuvastatin**	673900ATB, 674000ATB, 674100ATB
**Amlodipine+Rosuvastatin+Telmisartan**	671200ATB, 671300ATB, 671400ATB, 671500ATB, 677000ATB, 677100ATB, 671600ATB, 671700ATB
**Amlodipine+Telmisartan+Hydrochlorothiazide**	663500ATB, 663600ATB, 663700ATB, 663800ATB
**Amosulalol**	107901ATB, 107902ATB
**Arotinolol**	110202ATB, 110201ATB
**Atenolol**	483102ATB, 111402ATB, 483101ATB, 111403ATB
**Atenolol+Chlorthalidone**	262100ATB
**Azilsartan**	662401ATB, 662403ATB, 662402ATB
**Azilsartan+Chlorthalidone**	673500ATB, 673600ATB
**Barnidipine**	114003ACH, 114001ACH, 114002ACH
**Benidipine**	115101ATB, 115102ATB, 115104ATB, 115103ATB
**Betaxolol**	116801ATB, 116803ATB
**Bevantolol**	117002ATB, 117001ATB
**Bisoprolol**	117904ATB, 117903ATB, 117902ATB, 117901ATB
**Bisoprolol+Hydrochlorothiazide**	469800ATB, 470000ATB, 469900ATB

**Candesartan**	122601ATB, 122602ATB, 122603ATB
**Candesartan+Amlodipine**	652900ATB, 653000ATB, 653100ATB
**Candesartan+Hydrochlorothiazide**	423700ATB
**Candesartan+Rosuvastatin**	661800ATB, 661900ATB, 673700ATB, 662000ATB, 662100ATB
**Captopril**	122901ATB, 122902ATB, 122903ATB
**Captopril+Hydrochlorothiazide**	262200ATB, 262300ATB
**Carteolol**	124801ATB
**Carvedilol**	125005ATB, 125003ATB, 662201ATB, 125008ACR, 125001ATB, 662202ATB, 125007ACR, 125002ATB, 125006ACR, 125004ACR
**Celiprolol**	129101ATB
**Cilazapril**	133001ATB, 133002ATB, 133003ATB
**Cilnidipine**	133102ATB, 133101ATB
**Clonidine**	136505ATR
**Diltiazem**	145706ATB, 145707ACR, 145707ATR, 145703ACR, 145706ATR, 145707ATB
**Doxazocin**	149101ATB, 149102ATB, 149104ATR, 149103ATB
**Efonidipine**	441202ATB, 441201ATB
**Enalapril**	151603ATB, 151601ATB
**Enalapril+Hydrochlorothiazide**	453700ATB, 440300ATB
**Eprosartan**	429201ATB
**Eprosartan+Hydrochlorothiazide**	460500ATB
**Felodipine**	157503ATR, 157501ATR
**Felodipine+Metoprolol**	262400ATR
**Fimasartan**	515203ATB, 515201ATB, 515202ATB
**Fimasartan+Amlodipine**	651900ATB, 652000ATB, 652700ATB, 652100ATB
**Fimasartan+Hydrochlorothiazide**	522000ATB, 526800ATB
**Fimasartan+Rosuvastatin**	655000ATB, 654900ATB, 654800ATB, 654700ATB, 654600ATB
**Fosinopril**	163501ATB, 163502ATB

**Hydralazine**	170701ATB
**Imidapril**	173402ATB, 173401ATB
**Irbesartan**	177301ATB, 177303ATB
**Irbesartan+Atorvastatin**	524000ATB, 524100ATB, 527100ATB, 527000ATB
**Irbesartan+Hydrochlorothiazide**	385700ATB, 385800ATB, 553800ATB
**Lacidipine**	180301ATB, 180302ATB, 180303ATB
**Lercanidipine**	182001ATB, 182002ATB
**Lisinopril**	184501ATB
**Lisinopril+Hydrochlorothiazide**	499200ATB, 499300ATB
**Losartan**	185701ATB, 185702ATB
**Losartan+Amlodipine**	503000ATB, 637400ATB, 513900ATB, 637500ATB, 502700ATB, 637600ATB
**Losartan+Hydrochlorothiazide**	262500ATB, 486900ATB, 378900ATB
**Manidipine**	188001ATB, 188002ATB
**Metoprolol**	194003ATR, 193802ATB
**Metoprolol+Hydrochlorothiazide**	262600ATB
**Minoxidil**	196102ATB
**Nadolol**	198301ATB
**Nicardipine**	201003ACR, 201002ATB
**Nifedipine**	201407ACS, 201405ATR, 528201ATR, 201409ATR, 528202ATR, 201401ACS, 201401ATB, 201408ATR
**Nimodipine**	201901ATB, 356202ATR, 356203ATR, 356201ATB, 356202ATB
**Nisoldipine**	356202ATR
**Olmesartan**	468502ATB, 468501ATB, 468503ATB, 520902ATB, 520901ATB
**Olmesartan+Amlodipine**	547800ATB, 632800ATB, 500500ATB, 547700ATB, 629500ATB, 631300ATB, 500600ATB, 547900ATB, 632900ATB, 547600ATB, 548000ATB, 582200ATB, 629600ATB, 633000ATB, 547500ATB, 582400ATB, 629400ATB
**Olmesartan+Amlodipine+Hydrochlorothiazide**	519800ATB, 519700ATB, 520100ATB, 520000ATB, 519900ATB

**Olmesartan+Hydrochlorothiazide**	513600ATB, 489100ATB
**Olmesartan+Rosuvastatin**	644200ATB, 644100ATB, 526900ATB, 526300ATB, 526400ATB, 653200ATB, 526500ATB
**Perindopril**	211301ATB, 501601ATB, 211302ATB, 501602ATB
**Perindopril+Indapamide**	556200ATB
**Propranolol**	219901ATB, 219904ATB, 219906ACR, 219905ACR
**Quinapril**	221901ATB
**Ramipril**	222401ATB, 222402ATB, 222404ATB
**Ramipril+Felodipine**	447100ATB, 447200ATB
**Ramipril+Hydrochlorothiazide**	448600ATB, 448700ATB
**Telmisartan**	378801ATB, 378802ATB, 378803ATB
**Telmisartan+Amlodipine**	521200ATB, 511600ATB, 521300ATB, 511700ATB, 521400ATB, 511500ATB, 644800ATB, 623100ATB
**Telmisartan+Hydrochlorothiazide**	443200ATB, 443300ATB, 502600ATB
**Telmisartan+Rosuvastatin**	631600ATB, 629900ATB, 630000ATB, 631700ATB, 630100ATB, 630200ATB
**Temocapril**	235002ATB
**Terazosin**	235501ATB, 235502ATB, 235503ATB, 616501ATB
**Valsartan**	247103ATB, 247101ATB, 247102ATB, 247104ATB
**Valsartan+Amlodipine**	522600ATB, 492900ATB, 522900ATB, 523200ATB, 522700ATB, 492800ATB, 522800ATB, 523000ATB, 523300ATB, 495800ATB, 523100ATB, 523400ATB
**Valsartan+Hydrochlorothiazide**	356400ATB, 442600ATB
**Valsartan+Lercanidipine**	522200ATB, 522300ATB, 522400ATB
**Valsartan+Pitavastatin**	635000ATB, 635200ATB, 634900ATB, 635100ATB
**Valsartan+Rosuvastatin**	629700ATB, 525000ATB, 525200ATB, 629800ATB, 525100ATB, 525300ATB
**Valsartan+Sacubitril**	651401ATB, 651402ATB, 651403ATB
**Verapamil**	247606ATB, 247607ATB, 247603ATR, 247605ATR, 247601ACR
**Zofenopril**	510401ATB, 510402ATB, 510403ATB

**Atorvastatin**	111501ATB, 111502ATB, 111503ATB, 111504ATB, 502201ATB, 502202ATB, 502203ATB, 502204ATB
**Atorvastatin+Amlodipine**	472300ATB, 472400ATB
**Atorvastatin+Ezetimibe**	633800ATB, 633900ATB, 634800ATB
**Fluvastatin**	162401ACH, 162402ACH, 162403ATR
**Lovastatin**	185801ATB
**Pitavastatin**	470901ATB, 470902ATB, 470903ATB
**Pitavastatin+Fenofibrate**	679300ACH
**Pravastatin**	216601ATB, 216602ATB, 216603ATB, 216604ATB
**Rosuvastatin**	454001ATB, 454002ATD, 454002ATB, 454003ATB, 454003ATD, 454005ATB
**Rosuvastatin+Ezetimibe**	640700ATB, 640800ATB, 640900ATB
**Rosuvastatin+Ezetimibe+Telmisartan**	671400ATB, 671500ATB, 671700ATB
**Simvastatin**	227801ATB, 227802ATB, 227803ATB, 227805ATB, 227806ATB
**Aspirin**	110701ATB, 110702ATB, 110801ATB, 110802ATB, 110902BIJ, 111001ACE, 111001ATB, 111001ATE, 111002ATE, 111003ACE, 111003ATE
**Carbonyl iron**	367602ATB
**Chondroitin sulfate iron**	463501ACH, 463501AGN, 463502AGN
**Ferric chloride**	158501BIJ
**Ferric hydroxide polymaltose**	158601ALQ, 158602ALQ, 158603ALQ, 158608ALQ, 158630ALQ, 158631ALQ, 158632ALQ, 158633ALQ, 158634ALQ, 158635ALQ, 158636ALQ, 158637ALQ, 303300ATB
**Ferritinic iron**	417201ASY, 417230ASY
**Ferrous gluconate**	502800ALQ
**Ferrous sulfate**	303403ATR, 304700ALQ, 365700ALQ, 529200ALQ, 515100ASS
**Ferum oxide**	467001BIJ
**Iron acetyl transferrin**	379302ACH, 379301ALQ, 379330ALQ
**Iron dextran**	177501BIJ, 177530BIJ

**Iron hydroxide sucrose**	359701BIJ, 359702BIJ, 359730BIJ, 359731BIJ
**Iron proteinsuccinylate**	177601ALQ, 177630ALQ, 177602ATB
**Polysaccharide iron**	215001ACH, 215002ASY, 215005ASY, 215030ASY, 215031ASY, 215003ATB
**Sodium ferric gluconate**	228902ACH, 228903ALQ, 228904ALQ, 228930ALQ, 228931ALQ, 303800ALQ, 533700ALQ

Table S2. ICD-10 codes of Charlson Comorbidity Index

Comorbidities	Codes
**Myocardial infarction**	I21, I22, I252
**Congestive heart failure**	I43, I50, I099, I110, I130, I132, I255, I420, I425-I429, P290
**Peripheral vascular disease**	I70, I71, I731, I738, I739, I771, I790, I792, K551, K558, K559, Z958, Z959
**Cerebrovascular disease**	G45, G46, I60-69, H340
**Dementia**	F00-03, G30, F051, G311
**Chronic pulmonary disease**	J40-47, J60-67, I278-279, J701, J703, J684
**Rheumatologic disease**	M05-06, M32-34, M315, M351, M353, M360
**Peptic ulcer disease**	K25-28
**Mild liver disease**	B18, K73, 74, K700-703, K709, K713-715, K717, K760, K762-764, K768-769, Z944
**DM without complication**	E100-101, E106, E108-111, E116, E118-121, E126, E128-131, E136, E138-141, E146, E148-149
**DM with complication**	E102-105, E107, E112-115, E117, E122-125, E127, E132-135, E137, E142-145, E147
**Hemiplegia or paraplegia**	G81-82, G041, G114, G800, G830-834, G839
**Any malignancy**	C00-26, C30-C34, C37-41, C43, C45-58, C60-76, C81-88, C90-97
**Moderate to severe liver disease**	I850, I859, I864, I982, K704, K711, K721, K729, K765-767
**Metastatic tumor**	C77-80
**AIDS/HIV**	B20-22, B24.

Abbreviations: ICD-10, International Classification of Diseases, 10th revision, Clinical Modification; DM, diabetes mellitus; AIDS/HIV, acquired immune deficiency syndrome/human immunodeficiency virus

Table S3. Cox regression analyses using Groups 2 or 3 as reference groups

	Univariate analysis		Multivariable analysis	
	**Hazard ratio (95% CI)**	***p*-value**	**Hazard ratio (95% CI)**	***p*-value**
Group				
Ref: Group 2				
Group 3	1.22 (1.13–1.32)	<0.001	1.03 (0.93–1.13)	0.590
Group 4	1.32 (1.22–1.43)	<0.001	1.13 (1.02–1.25)	0.014
Ref: Group 3				
Group 4	1.08 (0.98–1.19)	0.102	1.11 (0.99–1.24)	0.087

Multivariable analysis is performed using an enter mode, with adjustment made for several variables, including sex; age; vascular access type; underlying cause of end-stage renal disease; hemodialysis vintage; Charlson Comorbidity index score; ultrafiltration volume; serum creatinine level; hemoglobin level; Kt/V_{urea}; serum albumin level; serum phosphorus level; serum calcium; systolic blood pressure; diastolic blood pressure; and use of anti-hypertensive drugs, aspirin, and statins; erythropoiesis stimulating agent dose; erythropoiesis stimulating agent resistance index; and use of iron; and was performed using enter mode. Group 1, patients with normal iron status; Group 2, patients with absolute iron deficiency; Group 3, patients with functional iron deficiency; and Group 4, patients with high iron status.

Table S4. Summary of previous studies evaluating the association between iron status and mortality in patients on HD using large sample sizes ($n \geq$ 10,000).

Author (year)	Data source	Primary goal of the study	Population	Main results for association between iron status and mortality
Kalantar-Zadeh K et al. (2005)	National database of DaviTa	To evaluate the association between iron status or supplementation and mortality	Incident and prevalent HD patients (*n* = 50,085)	Serum ferritin levels between 200 and 1200 ng/ml (reference 100 to 199 ng/ml), serum iron levels between 60 and 120 µg/ml (reference 50 to 59 µg/ml), and iron saturation ratio between 30 and 50% (reference 45 to 50%) were associated with the lowest all-cause and cardiovascular death risks.
Hatamizadeh P, et al. (2013)	National database of DaviTa	To evaluate optimal levels of iron status between HD patients with and without PKD	Incident and prevalent HD patients (2969 with PKD and 128,054 without PKD)	1. In patients with and without PKD, there was a U-shaped relationship between the average TSAT and mortality, and the best survival was shown in TSAT of 30–40%.<br>2. Best survival was shown with an average ferritin of 100–800 ng/mL in PKD patients and 500–800 ng/mL in non-PKD patients.
Kim T, et al. (2017)	National database of DaviTa	To evaluate the association between ferritin variation and mortality	Incident HD patients (*n* = 93,979)	Positive association between a major rise in serum ferritin in patients with a baseline ferritin ≥200 ng/mL or a slight rise in serum ferritin in those with a baseline ferritin ≥800 ng/mL during the first 6 months and mortality.
Karaboyas A, et al. (2018)	Data from the International Dialysis Outcomes and Practice Patterns Study	To evaluate the association between ferritin and mortality	Incident and prevalent HD patients (*n* = 18,261)	1. High ferritin levels were associated with elevated mortality (relative to region-specific medians) in all three regions (USA, Europe, and Japan).<br>2. The strength of the association between high ferritin levels and mortality was attenuated more by adjustment for malnutrition and inflammation than by IV iron and ESA dose.
Kuo KL, et al. (2018)	Taiwan Renal Registry Data System	To evaluate the association between anemia or iron status and mortality	Prevalent HD patients (42,230)	A serum ferritin level between 300 and 800 ng/mL and a TSAT value between 30% and 50% were associated with the lowest all-cause mortality.
Yeh SC, et al. (2019)	Taiwan Renal Registry Data System	To evaluate the association between iron indices and mortality in patients with and without PKD	1346 patients with PKD and 82,873 patients without PKD	1. The adjusted mortality risks for time-averaged ferritin levels >800 ng/mL (HR=1.52; 95% confidence interval: 1.40-1.65) or TSAT levels >50% (HR=1.46; 95% confidence interval: 1.30-1.65) were significantly higher among patients without PKD than those for patients with normal iron indices.<br>2. A U-shaped curve of mortality against ferritin/TSAT levels was not observed in patients with PKD.

Abbreviations: ESA, erythropoiesis-stimulating agent; HD, hemodialysis; IV, intravenous; PKD, polycystic kidney disease, TSAT, transferrin saturation rate.
